# Development and applicability of a quality control phantom for dental cone‐beam CT

**DOI:** 10.1120/jacmp.v12i4.3478

**Published:** 2011-11-15

**Authors:** Ruben Pauwels, Harry Stamatakis, Giorgos Manousaridis, Adrian Walker, Koen Michielsen, Hilde Bosmans, Ria Bogaerts, Reinhilde Jacobs, Keith Horner, Kostas Tsiklakis, The SEDENTEXCT Project Consortium

**Affiliations:** ^1^ Oral Imaging Center, Faculty of Medicine Katholieke Universiteit Leuven Leuven Belgium; ^2^ Department of Oral Diagnosis and Oral Radiology, School of Dentistry University of Athens Athens Greece; ^3^ Leeds Test Objects Ltd. Boroughbridge United Kingdom; ^4^ Department of Radiology University Hospitals Leuven Leuven Belgium; ^5^ Department of Experimental Radiotherapy University Hospitals Leuven Leuven Belgium; ^6^ School of Dentistry University of Manchester, Manchester Academic Health Sciences Centre Manchester United Kingdom

**Keywords:** cone‐beam computed tomography, dental equipment, quality control, phantoms, imaging, image processing, computer‐assisted

## Abstract

Cone‐beam CT (CBCT) has shown to be a useful imaging modality for various dentomaxillofacial applications. However, optimization and quality control of dental CBCT devices is hampered due to the lack of an appropriate tool for image quality assessment. To investigate the application of different image quality parameters for CBCT, a prototype polymethyl methacrylate (PMMA) cylindrical phantom with inserts for image quality analysis was developed. Applicability and reproducibility of the phantom were assessed using seven CBCT devices with different scanning protocols. Image quality parameters evaluated were: CT number correlation, contrast resolution, image homogeneity and uniformity, point spread function, and metal artifacts. Deviations of repeated measurements were between 0.0% and 3.3%. Correlation coefficients of CBCT voxel values with CT numbers ranged between 0.68 and 1.00. Contrast‐to‐noise ratio (CNR) values were much lower for hydroxyapatite (0<CNR<7.7) than for air and aluminum (5.0<CNR<32.8). Noise values ranged between 35 and 419. The uniformity index was between 3.3% and 11.9%. Full width at half maximum (FWHM) measurements varied between 0.43 mm and 1.07 mm. The increase of mean voxel values surrounding metal objects ranged between 6.7% and 43.0%. Results from preliminary analyses of the prototype quality control phantom showed its potential for routine quality assurance on CBCT. Large differences in image quality performance were seen between CBCT devices. Based on the initial evaluations, the phantom can be optimized and validated.

PACS numbers: 87.57.C‐, 87.57.N‐, 87.57.Q‐

## I. INTRODUCTION

Due to the increasing use of cone‐beam CT (CBCT) in dental practice and the large number of devices on the market, there is a need for a quantified and objective analysis of the technical image quality and radiation dose to enable an optimal use for this imaging modality.^(^ ^1^ ^,^ ^2^ ^)^ Three different aspects have to be considered in the optimization of an X‐ray imaging modality: quantification of the radiation dose and risk for patients, assessment of technical image quality, and assessment of diagnostic image quality. By means of an appropriate test object, the first and second aspect can be studied in one investigation process. Ideally, the development of test objects goes along with the formation of quality assurance (QA) protocols. During these activities, the diagnostic image quality must always be considered, implying that dose measurements are to be reported in terms of diagnostic needs, and technical image quality assessments need to be evaluated for their diagnostic relevance. This is particularly the case for dental imaging, as it involves a large variety of diagnostic indications requiring different imaging approaches.^(^ ^3^ ^)^

There is a lack of standardized tools for image quality analysis for dental CBCT. To develop such a tool, all available knowledge regarding image quality assessment on other 3D or pseudo‐3D imaging modalities (e.g., spiral CT, tomosynthesis, kV‐CBCT used in radiotherapy)^(^ ^4^ ^–^ ^7^ ^)^ needs to be combined with the existing knowledge of CBCT and previous studies on CBCT image quality.^(^ ^2^ ^,^ ^8^ ^–^ ^16^ ^)^ Even though a large number of CBCT image quality studies have been published over the last few years, most have focused on diagnostic image quality. However, a number of studies have already assessed technical image quality for one or more CBCT devices, using an existing commercial quality control (QC) phantom,^(^ ^8^ ^,^ ^9^ ^)^ a phantom provided by a CBCT manufacturer,^(^ ^2^ ^,^ ^10^ ^)^ a water phantom,^(^ ^8^ ^,^ ^11^ ^)^ a customized test object,^(^ ^12^ ^–^ ^15^ ^)^ or clinical data.^(^ ^16^ ^,^ ^17^ ^)^ Although these studies have provided useful insights regarding certain image quality aspects, they also show the need for a standardized QC phantom that is suited for use on all CBCT devices, and which provides results that are relevant to dental imaging and that can be compared between systems. Commercial QC phantoms have been described for conventional CT, but these are not applicable for dental CBCT due to the difference in performance for certain image quality aspects. CT phantoms use soft tissue‐equivalent materials for gray value analysis, which are not relevant for dental CBCT.^(^ ^6^ ^,^ ^7^ ^)^ Furthermore, dental imaging requires a high spatial resolution and a limitation of metal artifacts, both of which are not assessed by conventional CT phantoms.

A CBCT system uses a cone‐ or similarly shaped X‐ray beam that rotates around an object and acquires two‐dimensional projections reconstructed into a three‐dimensional volume.^(^ ^18^ ^)^ There are a variety of CBCT devices available with large differences for a number of imaging parameters: peak voltage, amount of filtration, quantity of X‐rays (mAs), pulsed versus continuous exposure, beam geometry, number of projections, detector type, field of view (FOV) size, reconstruction algorithm, reconstructed voxel size, and pre‐ and post‐processing of raw and reconstructed data. Designing a QC phantom requires a cross section of all available CBCT devices, identifying common properties. These properties, most of which are intertwined, are (ordered from general to specific): (1) CBCT images show very poor soft tissue differentiation, as they are meant for the visualization of hard tissues (bone, teeth) and air (sinus and air cavities); (2) spatial resolution is high (voxel sizes are generally below 0.4 mm) and nominally identical in all planes (isotropic); (3) most devices expose at a kVp below 100, and a low mAs; (4) there is a relatively large degree of scattered radiation resulting in image noise and nonuniformity; (5) voxel values are not standardized and cannot directly be used as quantitative CT numbers for use in bone mineral density (BMD) evaluation; (6) high‐density tissues and metal objects result in metal artifacts due to scatter, beam hardening, and photon starvation. All of these considerations affect the design of a QC phantom. Another limitation is the minimum FOV size of all currently available CBCT devices; the phantom must be suitable for all CBCTs, including those with a FOV of a few cubic centimeters.

The objective of the current study is to develop a quality control phantom that is suited for dental CBCT imaging, can be used on any CBCT device, and allows for the measurement of parameters which are relevant to dental imaging requirements. As an initial evaluation of the phantom, it was scanned using a variety of CBCT devices to evaluate the reproducibility and applicability of the evaluated parameters, and to investigate CBCT imaging performance.

## II. MATERIALS AND METHODS

### A. Development of quality control phantom

**Figure 1:**
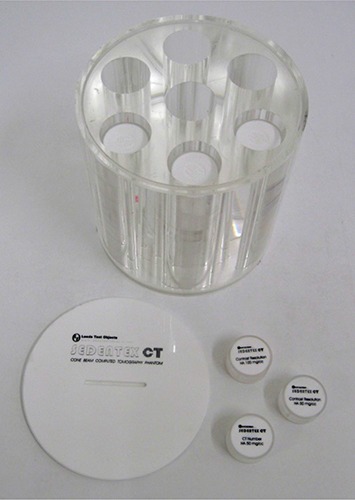
First prototype QC phantom and inserts.

The phantom was designed by Leeds Test Objects Ltd. in the frame of the EC project SEDENTEXCT (http://www.sedentexct.eu/). For the first prototype, a head‐size cylindrical polymethyl methacrylate (PMMA) phantom (160 mm diameter, 162 mm height) was designed with seven cylindrical holes positioned at the center and vertices of a regular hexagon (Fig. 1).

Along with this phantom, eight different cylindrical inserts (35 mm diameter, 20 mm height) were developed to test a total of six image quality parameters. A listing of currently evaluated image quality parameters, including the different materials and patterns that were used for each analysis, is shown in Table 1. For metal artifact analysis, titanium was selected because of the increasing use of titanium implants in dentistry.

**Table 1:** Listing of inserts developed for image analysis.

*Parameter*	*Insert Design*	*Image Analysis*
CT Number	Cylinders of five different materials (hydroxyapatite of varying density (50, 100 and $200\;\text{mg}.{\text{cm}}^{-3}$), aluminum, air) in PMMA surrounding	Average voxel value compared to Hounsfield Units obtained from MSCT scanners in correlation plot
Contrast Resolution	Same as CT number	Contrast‐to‐noise ratio calculation using central material and surrounding PMMA
Image Homogeneity	PMMA inserts	Normalized standard deviation
Image Uniformity	PMMA inserts	Difference in average voxel value between insert columns
Point Spread Function	Steel wire (0.25 mm) suspended in air	1D integrated full width at half maximum calculation
Metal Artefacts	Three in‐line titanium rods	Increase of voxel values in vicinity of rods

### B. Scanning of the phantom and inserts on CBCT and MSCT systems

To evaluate the reproducibility of the evaluated parameters before applying them to scans of various CBCT devices, the phantom and inserts were scanned five consecutive times using the SCANORA 3D CBCT (Soredex, Tuusula, Finland), with a clinical standard resolution protocol as depicted in Table 2. Measurements of all image quality parameters described below were performed on each scan. Also, measurements were repeated five times for one scan, to evaluate the reproducibility of the measurement itself.

**Table 2:** CBCT and spiral CT scan parameters.

*CBCT*				
	*Field Size (mm)* ^a^	*Tube Potential (kV)*	*mAs*	*Voxel Size (mm)*
GALILEOS Comfort	150 × 150	85	28	0.3
i‐CAT Classic High‐dose	160 × 80	120	35	0.2
i‐CAT Classic Low‐dose	160 × 80	120	10	0.4
ILUMA Elite	210 × 140	120	76	0.2
ProMax 3D High‐dose	80 × 80	84	168	0.16
ProMax 3D Low‐dose	80 × 80	84	20	0.32
SCANORA 3D High‐dose	60 × 60	85	36	0.13
SCANORA 3D Low‐dose	60 × 60	85	24	0. 2
SCANORA 3D Reproducibility	145 × 75	85	24	0.35
SkyView High‐dose	173 × 173	90	96	0.34
SkyView Low‐dose	173 × 173	90	52	0.34
Veraviewepocs 3D	80 × 80	70	51	0.13

*Spiral CT*				
	*Field Size (mm)*	*Tube Potential (kV)*	*mA*	*Slice Thickness (mm)*
GE Prospeed	N/A	120	200	1
Siemens SOMATOM Sensation 64	N/A	80/100	208/199	1/2.5

a diameter×height

Subsequently, the inserts were scanned on seven CBCT devices: GALILEOS (Sirona Dental Systems, Bensheim, Germany), i‐CAT Classic (Imaging Sciences International, Hatfield, PA, USA), ILUMA Elite (IMTEC, Ardmore, OK, USA), ProMax 3D (Planmeca Oy, Helsinki, Finland), SCANORA 3D (Soredex, Tuusula, Finland), SkyView (MyRay, Imola, Italy), Veraviewepocs 3D (J. Morita, Kyoto, Japan). Exposure parameters for all devices can be found in Table 2. All selected protocols are used in clinical practice.

**Figure 2:**
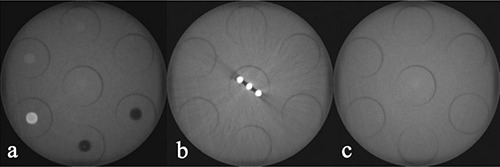
Axial slices of SCANORA 3D phantom scan, using the large FOV: (a) top row containing CT number / contrast resolution inserts and point spread function insert; (b) middle row with metal artifact insert; (c) bottom row containing PMMA inserts.

All inserts were placed in the PMMA holder phantom for scanning. For all scanners with a small‐ or medium‐sized field of view (FOV) that were unable to scan the entire phantom, the insert of interest was positioned in one of the peripheral holes to mimic an actual dental scan. The inserts were scanned centrally in the FOV. Holes not containing the insert were filled up using blank PMMA inserts. For scanners with a large FOV (~15 cm diameter or more), inserts were positioned at the six peripheral positions of the holder phantom. The holder phantom was scanned centrally in the FOV, to mimic a full head scan. For all scanners, three insert rows were used: the bottom row contained blank PMMA inserts, the middle row contained the metal artifact insert, and the top row was used for all other inserts (Fig. 2). An exposure used for a standard adult patient was selected for each CBCT. Whenever possible, high‐ and low‐dose clinical protocols were selected by varying the mAs. For CBCT, high‐dose protocols typically imply a smaller voxel size owing to a modified reconstruction.

In addition to CBCT scans, spiral CT scans were acquired from the inserts to obtain CT number measurements serving as the gold standard. Three scanning protocols were used from two different scanners: GE Prospeed (General Electric, Fairfield, CT, USA) and SOMATOM Sensation 64 (Siemens AG, Erlangen, Germany) (Table 2).

### C. Analysis of image quality inserts

All datasets were evaluated with the ImageJ software (National Institutes of Health, USA) using a combination of different image analysis tools. For all measurements except point spread function, the measurement was performed on ten consecutive axial slices to obtain a sufficient sample size. Measured parameters (mean voxel value, standard deviation) were averaged over these slices. The top and bottom of the insert were avoided because of possible interference by adjacent inserts.

#### C.1 CT number correlation and contrast resolution

The inserts for CT number evaluation, containing rods of five different densities, were analyzed by measuring the mean voxel value obtained from circular ROIs along different axial slices through the insert. Apart from the five materials involved in the insert, the voxel value from PMMA was obtained using a blank insert. From all six materials, corresponding CT numbers were obtained from spiral CT scans by taking the average value from the three CT protocols that were applied. Correlation coefficients were determined for a linear fit.

**Figure 3:**
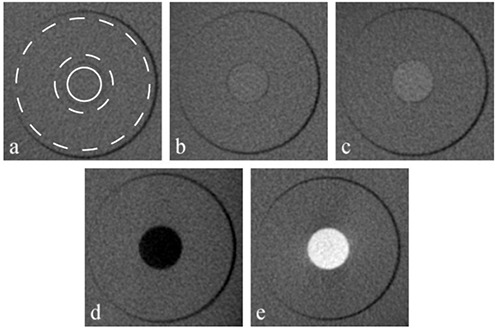
Contrast resolution inserts, containing five rods of different materials: (a) hydroxyapatite $50\;\text{mg}/{\text{cm}}^{3}$; (b) hydroxyapatite $100\;\text{mg}/{\text{cm}}^{3}$; (c) hydroxyapatite $200\;\text{mg}/{\text{cm}}^{3}$; (d) air; (e) aluminum. The ROIs used for the material (solid circle) and PMMA background (area between dashed circles) are shown in (a).

The same inserts were used to determine the contrast‐to‐noise ratio (CNR) for the five different materials. To calculate the CNR, circular regions of interest (ROI) were positioned on the inner part of the materials and on the adjacent PMMA (Fig. 3). The mean voxel value and standard deviation were determined, and the CNR was calculated using the formula:

(1) $$\text{CNR}=\frac{{\text{MVV}}_{m}-{\text{MVV}}_{b}}{{\text{SD}}_{m,b}}$$

where ${\mathrm{MVV}}_{m}$ and ${\mathrm{MVV}}_{b}$ are the mean voxel values for the evaluated material and the (PMMA) background, respectively, and $SD_{m,b}$ is the average standard deviation of the voxel values within the material and background.

#### C.2 Homogeneity and uniformity

Blank PMMA inserts were used to evaluate image homogeneity and uniformity. The noise was determined by measuring the standard deviation of voxel values within a blank PMMA insert. It was chosen not to calculate a signal‐to‐noise ratio (SNR), since voxel values of homogeneous PMMA do not represent the amount of signal. It was seen that most CBCT datasets use a conventional 12‐bit scale, but some use a higher or lower bit depth which makes it impossible to compare standard deviations of voxel values. Therefore, the measured values were converted to a 12‐bit scale where needed. Uniformity of voxel values in the XY‐plane was determined for large‐volume scanners by filling the central and peripheral holes of the phantom with blank PMMA inserts and positioning the phantom centrally in the FOV, thus yielding seven ROIs. For small‐volume scanners, the FOV was positioned on a peripheral PMMA insert, and the adjacent areas were used as peripheral ROIs. All measurements of voxel values were converted to a 12‐bit scale with the lowest possible voxel value being 0. The uniformity parameter was defined as the percentage of mean voxel value difference between the areas with the highest and lowest value.

#### C.3 Point spread function

**Figure 4:**
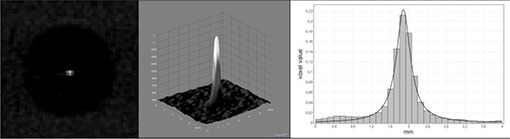
Point spread function of 0.25 mm wire, showing an example axial slice (left), a 2D surface plot from a small central ROI (middle), and an integrated one‐dimensional profile fitted to a Cauchy distribution (right).

The point spread function (PSF) insert was evaluated by determining the 2D profile from the wire and surrounding air, and integrating this along the y‐axis to yield a one‐dimensional PSF. The resulting distribution was fitted to a Cauchy distribution (i.e., Lorentz distribution, a continuous probability distribution similar to the Student's t‐distribution), using EasyFit 5.0 (MathWave Technologies, Dnepropotrovsk, Ukraine), enabling the determination of the full width at half maximum (FWHM) value (Fig. 4).

#### C.4 Metal artifacts

**Figure 5:**
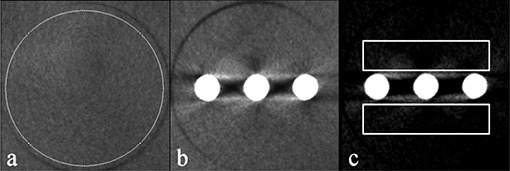
Consecutive steps in streak artifact measurement: (a) measurement of PMMA mean voxel value; (b) example axial slice of metal artifact insert; (c) after subtraction of PMMA mean voxel value, showing regions of interest.

For measuring the extent of metal artifacts, the average voxel value from the PMMA insert was subtracted from the scan of the metal artifact insert. Subsequently, the average voxel value was measured at two ROIs surrounding the titanium rods. All steps are illustrated in Fig. 5.

The ‘artifact added value’ was then defined as:

(2) $$\text{AAV}=\frac{{\text{MVV}}_{\text{art}}+{\text{MVV}}_{\text{PMMA}}}{{\text{MVV}}_{\text{PMMA}}}$$

where ${\mathrm{MVV}}_{\mathrm{art}}$ and ${\mathrm{MVV}}_{\mathrm{PMMA}}$ are the mean voxel values for the artifact after subtraction and the blank PMMA insert, respectively. The *AAV* is independent of factors such as noise and bit depth. Its value is mainly defined by those parts of the metal artifact which show higher values than that of PMMA, seeing that all values below that of PMMA are subtracted to the lowest possible voxel value. The value of AAV increases when white streaks originating from the metal object cover a larger area or when they are more pronounced.

## III. RESULTS

### A. Reproducibility of image quality parameters

Average and maximal deviations of repeated measurements obtained from five consecutive scans using an identical exposure and five repeated measurements on a single dataset are shown in Table 3. This deviation represents the interscan and intrascan reproducibility of the phantom and measuring methods. For the different materials involved in contrast analysis, the measurement of mean voxel value was used rather than the CNR. The average deviation for all investigated parameters was 1.3% for consecutive scans and 1.0% for measurements on an identical scan. The highest deviations were seen for the measurements of hydroxyapatite (2.2%–3.3%) and the FWHM of the point spread function (2.0%–2.7%). The highest reproducibility was seen for the CT number correlation coefficient (0.01%–0.02%).

**Table 3:** Reproducibility of repeated measurements on consecutively scanned (inter) and identical (intra) CBCT datasets.

	*Average Deviation (%)*		*Maximal Deviation (%)*	
	*Inter*	*Intra*	*Inter*	*Intra*
Air	0.2	0.2	0.4	0.4
PMMA	0.7	0.4	1.7	0.7
HA50	2.2	1.2	5.6	2.7
HA100	3.3	2.6	5.9	6.8
HA200	2.8	2.7	4.3	4.6
Aluminum	0.5	0.3	0.8	0.5
Artifacts	1.1	0.5	2.2	1.4
Noise	0.3	0.4	0.8	0.9
Uniformity	0.8	0.7	1.6	1.7
FWHM	2.7	2.0	5.2	4.2
CT Number Correlation	0.0	0.0	0.0	0.0
Average of All Parameters	1.3	1.0	2.6	2.2

### B. CT number and contrast resolution

Example slices from scans obtained from the five different inserts used for CT number and contrast analysis are depicted in Fig. 3. Table 4 shows the correlation coefficients between CT numbers measured on spiral CT scans and voxel values measured on CBCT scans. Correlation coefficients ranged between 0.6864 and 0.9996 (average: 0.9689) when including all materials. For the medium density range (hydroxyapatite 50,100 and $200\;\text{mg}/{\text{cm}}^{3}$ and PMMA), the correlation coefficient ranged between 0.7303 and 0.9909 (average: 0.9156).

**Table 4:** Image analysis results for CBCT scanners.

*Device*	$CNR_{HA50}$	$CNR_{HA100}$	$CNR_{HA200}$	${\mathrm{CNR}}_{\mathrm{AIR}}$	${\mathrm{CNR}}_{\mathrm{AL}}$	${\mathrm{CT}}_{\mathrm{ALL}}$	${\mathrm{CT}}_{\mathrm{MED}}$	*AAV (%)*	*Noise*	*Uniformity (%)*	*FWHM (mm)*
GALILEOS Comfort	0.0	1.0	1.0	5.0	8.7	0.9980	0.8159	14.3	89.4	8.1	0.57
i‐CAT Classic High‐dose	1.7	2.6	3.8	15.0	21.0	0.9973	0.9070	19.2	69.1	11.8	0.49
i‐CAT Classic Low‐dose	0.4	2.6	4.4	17.3	21.2	0.9870	0.9661	24.3	56.6	21.1	0.97
ILUMA Elite	0.0	0.0	0.8	13.6	15.0	0.9946	0.7303	6.7	82.6	3.3	0.70
ProMax 3D High‐dose	0.0	0.4	2.1	11.4	19.9	0.9996	0.9614	23.4	70.6	11.9	0.77
ProMax 3D Low‐dose	0.0	0.1	1.0	6.5	10.8	0.9993	0.9236	24.0	126.5	11.2	0.97
SCANORA 3D High‐dose	0.5	0.8	2.0	8.9	9.4	0.9988	0.9909	11.6	81.6	7.9	0.43
SCANORA 3D Low‐dose	0.0	0.9	1.7	9.4	9.5	0.9993	0.9781	11.2	80.6	13.5	0.45
SkyView High‐dose	2.5	4.1	7.7	19.2	32.8	0.9990	0.9679	40.2	35.1	4.0	0.68
SkyView Low‐dose	2.2	3.5	7.3	16.5	28.5	0.9986	0.9719	43.0	41.2	4.9	0.71
Veraviewepocs 3D	0.6	1.3	1.6	6.4	13.0	0.6864	0.8581	9.4	418.6	6.4	1.07

CNR=contrast‐to‐noise ratio; $CT_{ALL}=$ overall correlation coefficient with CT numbers; ${\text{CT}}_{\text{MED}}=$ correlation coefficient for medium densities only; AAV= artifact added value; Noise= standard deviation of PMMA voxel values; FWHM= full width at half maximum

Table 4 depicts CNR values obtained from the inserts. Contrast was shown to be low in general for all three hydroxyapatite densities (0<CNR<7.7), and high for aluminum and air (5.0<CNR<32.8), showing a large range between the different CBCT devices and protocols.

### C. Homogeneity and uniformity

Table 4 shows noise values for all CBCT datasets. Large differences are seen between devices, with standard deviations of voxel values ranging between 35 and 419. When considering high‐and low‐dose protocols, it is shown that there is no clear correlation between dose and noise for different CBCT devices, given that the images are reconstructed using different voxel sizes.

Uniformity index calculations also vary between devices (Table 4). Generally, uniformity is better for high‐dose scans.

### D. Point spread function

Table 4 contains FWHM values, which range between 0.43 mm and 1.07 mm, showing clear differences between high‐ and low‐resolution scans of certain CBCT devices.

### E. Metal artifacts

**Figure 6:**
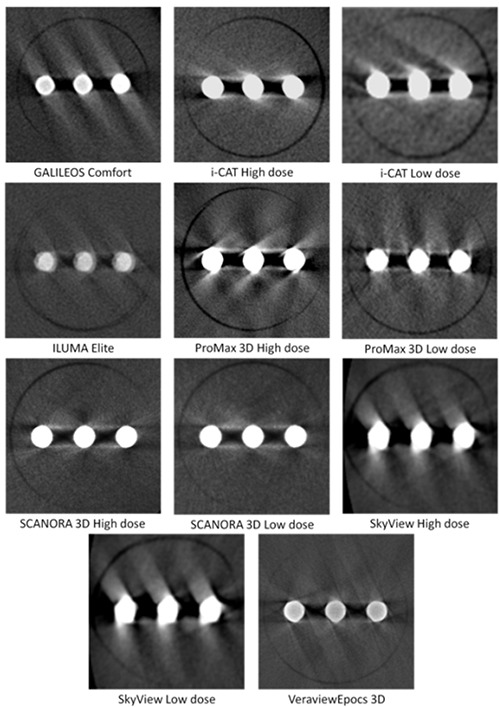
Example axial slices for metal artifact inserts, showing three titanium rods.

Example slices of the metal artifact insert for each are shown in Fig. 6. Table 4 shows results for the artifact added value (AAV), which ranged between 10.4 and 40.6. No clear difference is seen for high‐ and low‐dose protocols of any device (p<0.05 for Wilcoxon signed rank test).

## IV. DISCUSSION

In this study, the applicability of a first prototype quality control phantom for CBCT was evaluated. Reproducibility of the measurements of image quality parameters was assessed, and the range of values obtained from all parameters was investigated for a wide range of devices and exposure protocols to assess the applicability of the selected insert designs, materials, and image analysis methods.

The design of the phantom was tailored for its application on dental CBCT devices. A head‐sized PMMA cylinder was selected to ensure a simplified yet proper simulation of the average attenuation of the head. PMMA is a standard material used for dosimetric and image quality phantoms.^(^ ^5^ ^–^ ^7^ ^,^ ^9^ ^,^ ^10^ ^,^ ^12^ ^,^ ^15^ ^)^ Although the human head contains a number of air cavities — most notably the oral cavity and sinuses — there are a number of high‐density structures as well, such as the temporal bone, mandibular cortical bone, and tooth enamel. Therefore, the PMMA phantom used in this study can be considered to result in detector photon fluencies similar to the human head. As a result, the different technical image parameters measured using this phantom can be related to the CBCT devices' clinical performance.

Furthermore, all selected materials for image quality evaluation are relevant for dental imaging. For contrast resolution and voxel value analysis, different densities of hydroxyapatite were selected to represent various bone densities, and aluminum was used to represent dense cortical bone. For metal artifact evaluation, titanium rods resembling dental implants were used, bearing in mind that titanium implants are major contributors to image quality degradation for dental CBCT.^(^ ^19^ ^)^ The nominally high spatial resolution of CBCT devices was assessed by choosing a thin wire to estimate the point spread function. By using small size inserts, the phantom can be applied to all CBCT devices, as the smallest FOV of all CBCT devices on the market (40×30 mm) is larger than the insert size (35×20 mm). Furthermore, placing these inserts peripherally in the phantom is clinically relevant, seeing that almost all tissues that are investigated in dentomaxillofacial imaging are found peripherally in the head (teeth, jaw bones, sinuses, temporomandibular joint). It is especially important for CBCT devices with small FOVs to mimic this clinical situation as it has been shown that the size and position of a FOV can affect the image quality for these devices.^(^ ^12^ ^,^ ^14^ ^)^

Another specific issue in CBCT imaging is the wide cone angle used by some devices, which leads to image quality degradation and artifacts. These artifacts may occur at the top or bottom of the FOV, and result in voxel values that are unsuitable for image quality analysis. However, seeing that three rows of inserts were used, the part of the phantom that was used for image analysis was limited to 6 cm in height. Furthermore, for those scanners with a small FOV height, insert rows were scanned separately to avoid these artifacts for the top and bottom inserts. In addition, measurements of all image quality parameters were performed in slices around the center of the height of the insert, avoiding any interference that may occur at the border between adjacent inserts.

It was possible to accurately position and scan the phantom on the different CBCT devices with supine, seated, and standing patient positioning. The total time needed to obtain a full phantom dataset depended largely on the FOV size. For some devices, a single scan sufficed to include all inserts; for others, it was necessary to scan each insert separately, leading to an increase in the total time needed to obtain a full set of scans of the phantom. This total time is a summation of the time needed to position the phantom, the lag time between consecutive scans, and the time needed to reconstruct and export datasets. All these factors are affected by the available FOV size. This implies that the insert size, and also the number of inserts, is limited by the small‐volume CBCTs, as it is not practical to perform a large number of scans for an image quality QC procedure. For further prototyping, we will investigate whether the number of inserts can be lowered by combining the different materials used for contrast resolution analysis into a single insert.

All measurements of image quality parameters were proven to be reproducible for consecutive scans, as well as for identical scans. It can be expected that consecutive scans differ slightly in terms of voxel values due to slight variability in tube output. This was generally reflected in the measurements, seeing that the deviations on consecutive scans were typically larger than for repeated measurements on a single scan, which shows the sensitivity of the image quality assessment for small variations of image quality. The largest variability was found for low‐contrast resolution, which is susceptible for slight variations in voxel value or noise measurements, because the low‐contrast resolution for dental CBCT is generally poor. Variability was also seen for the point spread function analysis, most likely due to undersampling which is of specific concern for low‐resolution datasets. An image analysis technique using oversampling can increase the accuracy of the FWHM estimation.^(^ ^10^ ^)^

For most devices, there was a good overall correlation between voxel values from different materials and CT numbers obtained from spiral CT scanners, but the correlation for medium‐density materials was worse. Two important factors contribute to this finding. First, voxel values are affected by the amount of mass outside the reconstructed volume, since this mass results in variable projection data due to different amounts of scattered radiation. This is especially the case for small‐volume CBCTs. Katsumata et al.^(^ ^14^ ^)^ showed a relation between density variability and imaging volume, and the present study is in accordance with those findings. Secondly, the amount of mass within the FOV may affect the gray value distribution, depending on the reconstruction algorithm used. Therefore, the presence of high‐ or low‐density materials in the FOV can affect the distribution of voxel values. Similar findings were reported by Bryant et al.^(^ ^12^ ^)^ In contrast to the current results, Lagravère et al.^(^ ^15^ ^)^ and Naitoh et al.^(^ ^17^ ^)^ have stated that a linear conversion can be made between density measurements on a CBCT scan and CT numbers. Furthermore, density measurements were independent of the position in the volume.^(^ ^15^ ^)^ However, one must take care when applying statistical analysis methods to this type of measurement, as a statistical significance or insignificance can be difficult to interpret. In order to claim an accurate and stable relation between CT numbers and CBCT voxel values, there is not only the requirement of a high correlation, but CBCT values also need to be independent of exposure and positioning factors to allow rescaling to CT numbers. The presented results do not support this claim for the investigated devices.

Contrast measurements further confirmed the general poor contrast between materials of intermediate density. CNR measurements showed that the noise is often similar or larger than the measured difference in mean voxel value between low‐density hydroxyapatite and PMMA. For air and aluminum, large differences were seen for CNR values between devices.

The present study showed that image homogeneity and uniformity values were not only affected by the exposure. The reconstructed voxel size, the size of the imaging field, and the amount of mass outside the volume were additional parameters leading to differences in device performance. Bryant et al.^(^ ^12^ ^)^ have defined the “exo‐mass” effect as the gradient of voxel values appearing for asymmetrical phantom positioning, leading to a shift in density response throughout the entire volume, as well as a decrease in uniformity. This results in different noise and uniformity values for small‐ and large‐field CBCTs.

A general consistency could be observed between FWHM values obtained from the point spread function and voxel sizes of the CBCT images. However, it can be clear that the voxel size itself provides only a crude prediction of the spatial accuracy.^(^ ^2^ ^,^ ^16^ ^)^ The voxel size, determined by the manufacturer, should ideally provide a balance between spatial resolution and noise. It is seen that for some devices, predefined exposure protocols using different mAs values are reconstructed at different voxel sizes. As seen from the FWHM measurements, high‐dose protocols showed an increased spatial resolution in most cases. Two exceptions were found, for which there was no clear difference in FWHM values between exposure protocols from the same device. For these exceptions, high‐ and low‐dose protocols were reconstructed at similar or identical voxel sizes, and the increase in mAs did not result in an improved spatial resolution. It must be noted that the point spread function can be influenced by a number of additional factors such as the difference in voxel values for the steel wire and the surrounding air, and the presence of small streak artifacts.

Metal artifacts from titanium rods appear different when comparing CBCT devices and protocols. A method was established to quantify streaks by subtracting background voxel values and calculating an artifact added value (AAV). The AAV provides a relevant description of the net effect of streaks on image quality, but provides no information regarding beam hardening and photon starvation artifacts. Imai et al.^(^ ^20^ ^)^ proposed a statistical analysis of streak artifacts on CT images using the extreme value theory. This approach may provide a partial solution regarding a full artifact analysis for CBCT.

From a QC perspective, the variability of all image quality parameters show that a general threshold or range for acceptable parameter values cannot be established for CBCT, and quality control should be based on the initial performance of the device at the time of installation and acceptance testing. These initial image quality results would then serve as baseline values for further tests on this particular device. Large deviations of QC results compared with the initial performance would then point out that the performance of the device needs to be assessed by the equipment installer or the manufacturer, or that the device should be temporarily suspended from clinical use until the performance issue has been fixed.

In addition to this type of baseline QC evaluation, it could be possible to establish specific image quality criteria for CBCT. To enable this, an evaluation of the diagnostic relevance is required for all technical image quality parameters to establish a QA protocol based on these parameter values. For some parameters, this relevance is obvious, but their true diagnostic effect is not always clear. Based upon a poor correlation between voxel values and CT numbers, along with a high degree of noise and poor image uniformity, it may be concluded that CBCT images are not useful for quantitative analysis of bone quality. Bone analysis methods do not, however, necessarily rely on absolute or relative voxel values.^(^ ^21^ ^)^ The results demonstrate that a CT number correlation analysis should not be part of a QC procedure for dental CBCT, as many manufacturers do not claim to have an accurate correlation with CT numbers for their devices. Nevertheless, these devices can still be used in clinical practice for analysis of bone quality. As another example, a high contrast between aluminum and PMMA can partly predict a good bone segmentation quality *in vivo*, but the latter also depends on factors such as geometric accuracy and spatial resolution of the system, artifacts originating from the cortical bone, and other high‐attenuation objects such as implants and metallic restorations.^(^ ^16^ ^)^ The further development of the QC phantom and the formation of a QA protocol will therefore be conducted in parallel with research on diagnostic image quality, which could allow determination of specific ranges or thresholds for certain image quality parameters. Our results suggest that different ranges may have to be applied for large and small FOV devices, since they perform differently in terms of noise and uniformity.

There are a number of possible improvements for the phantom, which will be implemented and evaluated in further development stages. Regarding the choice of materials, the results indicate that low‐density hydroxyapatite is not a suitable material for contrast evaluation due to the poor performance for most CBCT devices in terms of low‐contrast resolution. Alternative materials will be investigated. Furthermore, the analysis of point spread function and metal artifacts will be fine‐tuned. The measurements of the FWHM would be more accurate if an oversampling technique is used.^(^ ^10^ ^)^ Also, the current phantom provides no analysis of spatial resolution along the z‐axis, as the FWHM estimation is performed in the axial (x‐y) plane. Although CBCT datasets are nominally isotropic, it is worthwhile to verify this by measuring the spatial resolution along all axes. The analysis of metal artifacts used in the current study provides a useful estimation of the artifact's effect on the adjacent region, but should be altered to involve the entire insert rather than a fixed region of interest. Furthermore, the image quality parameter used for artifacts should be independent of actual voxel values, as these values are not standardized in CBCT imaging. Further in‐depth study is required to determine the ideal image analysis method for metal artifacts.

The measurement of radiation dose was not included in the current study, even though a comparison of high‐ and low‐dose protocols provided certain insights concerning the relation between image quality and radiation dose. To establish a correct definition of image quality, there is a need for an accurate but relatively simple method for estimating the corresponding radiation dose using routine measurements. These measurements then need to be related to patient risk. Existing methods do not cope well with the exposure geometry and dose distribution of CBCT.^(^ ^22^ ^,^ ^23^ ^)^ The development of a suitable dose index will establish the possibility for an extensive evaluation of CBCT performance, optimizing image quality versus radiation dose.

## V. CONCLUSIONS

The studied phantom has shown promising potential for technical image quality evaluation of CBCT. Different image quality parameters were measured for a wide range of CBCT devices and protocols. Future work should focus on optimizing the phantom and insert design, while establishing a QA protocol with appropriate criteria or ranges for each image quality parameter.

## ACKNOWLEDGMENTS

The research leading to these results has received funding from the European Atomic Energy Community's Seventh Framework Programme FP7/2007‐2011 under grant agreement no 212246 (SEDENTEXCT: Safety and Efficacy of a New and Emerging Dental X‐ray Modality). The Manchester author acknowledges the support of the NIHR Manchester Biomedical Research Centre.
